# Potential and Pitfalls of Digital Voice Assistants in Older Adults With and Without Intellectual Disabilities: Relevance of Participatory Design Elements and Ecologically Valid Field Studies

**DOI:** 10.3389/fpsyg.2021.684012

**Published:** 2021-07-01

**Authors:** Anna Schlomann, Hans-Werner Wahl, Peter Zentel, Vera Heyl, Leonore Knapp, Christiane Opfermann, Torsten Krämer, Christian Rietz

**Affiliations:** ^1^Network Aging Research, Heidelberg University, Heidelberg, Germany; ^2^Institute for Educational Sciences, Heidelberg University of Education, Heidelberg, Germany; ^3^Institute of Psychology, Heidelberg University, Heidelberg, Germany; ^4^Institute of Prevention, Integration and Rehabilitation Research, Ludwig-Maximilians-Universität Munich, Munich, Germany; ^5^Institute for Special Needs Education, Heidelberg University of Education, Heidelberg, Germany

**Keywords:** speech assistant, smart speaker, aging, old age, digitization, ecological validity, digital diary

## Introduction

While digital voice assistants (VAs) are increasingly becoming part of everyday life (Auxier, 2019), specific user groups that might benefit from such technology are often not considered in research. This applies to older adults (Stigall et al., 2019), a population with pronounced heterogeneity in terms of cognitive, sensory and functional competencies. Given the rapid expansion of digital tools, it is important to consider the use of VAs as a promising tool able to maintain and enhance social participation, autonomy, and leisure activities for a wide range of older adults. There is still a digital divide between younger and older generations (Anderson, 2019) and older adults might benefit to a smaller extent from the advantages of new technologies such as VAs. Older people with intellectual disabilities are at particular risk of digital exclusion (Ehlers et al., 2020) and digital technologies such as VAs have hardly been evaluated for this target group (e.g., Smith et al., 2020). In this opinion paper, we synthesize current research in the context of VAs for older adults. Building on this, we propose specific research designs to provide better insights into the adoption and use of VAs in advanced age.

## Benefits of Voice Assistants

An overview of literature provides six clusters of insights. First, VAs offer technology access for individuals who do not use conventional computing devices. *Usability* problems caused by small font or buttons are eliminated (Ziman and Walsh, 2018; Kowalski et al., 2019; Corbett et al., 2021), which is especially useful for individuals with limited motor, sensory, or cognitive functions (e.g., Yaghoubzadeh et al., 2013; Wulf et al., 2014). Second, in the *social domain*, VAs enable contact and communication with others, especially for older people with disabilities like limited vision or impaired hand movement (Kowalski et al., 2019; Scherr et al., 2020; Trajkova and Martin-Hammond, 2020). Additionally, the VA itself can be a social companion to some extent (Scherr et al., 2020; Smith et al., 2020; Corbett et al., 2021). Third, a benefit has been identified in the *health domain*. VAs can assist with daily well-being activities such as health tracking, medication management, or dietary planning (Tsiourti et al., 2014; Nallam et al., 2020; Trajkova and Martin-Hammond, 2020). Fourth, the use of VAs might contribute to enjoyable *leisure time* experiences, via entertainment features like music, videos, and jokes (Scherr et al., 2020; Corbett et al., 2021). Fifth, VAs can provide *support for independent living* including aspects of time-structuring (e.g., setting a timer or reminders) and instrumental activities like access to online information (Nallam et al., 2020; Pradhan et al., 2020; Scherr et al., 2020; Corbett et al., 2021). Sixth, taken together, VAs may have a positive effect on a person's *agency* because VAs can support older adults with intellectual disabilities in better managing different aspects of their everyday lives (Smith et al., 2020).

## Challenges of Voice Assistants and Limitations in Current Research on Voice Assistants in Older Adults

Although it boasts good usability in general, *problems interacting* with VAs are frequently observed, because the user is required to follow a pre-structured form of dialogue, thus limiting the conversational abilities of VAs (Scherr et al., 2020). Older adults often have problems recalling the specific commands necessary to operate the devices (Wulf et al., 2014; Pradhan et al., 2020). Another limiting factor can be the *lack of added value*, which may result in a preference for devices already used (Trajkova and Martin-Hammond, 2020). VAs are perceived as time consuming and a *lack of compatibility* is criticized (Kowalski et al., 2019). Furthermore, a barrier for using VAs can be seen in reported fear of *losing one's own competences and autonomy*, because the VA may take care of a number of tasks without considering the competencies of the user (Kowalski et al., 2019; Trajkova and Martin-Hammond, 2020). In this way, the benefit of support for independent living and a reduction of dependency on personal assistance may also result in a *higher level of dependency* on VA assistance. Finally, concerns about *privacy and data security* are potential barriers to using VAs (Nallam et al., 2020; Trajkova and Martin-Hammond, 2020).

On a more general level, two major limitations in research on VAs for older adults exist. There is limited knowledge on VA use in *specific groups of older adults*. We identified two studies that focus on benefits and challenges for older adults with cognitive impairment (e.g., dementia; Wargnier et al., 2015; Wolters et al., 2016) and two other studies that included older people with intellectual disabilities (Braun et al., 2020; Smith et al., 2020). We also see *limitations at the methodological and design level*. In particular, we identified six studies that apply field data collection—collecting data in everyday life settings to analyze the use of VAs among older adults (Tsiourti et al., 2014; Kopp et al., 2018; Oh et al., 2020; Pradhan et al., 2020; Scherr et al., 2020; Corbett et al., 2021). Similarly, user-centered research approaches have been infrequently applied thus far.

## Recommendations For Future Research

Based on these insights, we derive recommendations for research on VAs (see Table 1). We put emphasis on subgroups of older adults who have been largely neglected, i.e., older adults with cognitive impairment like dementia-related disorders or intellectual disabilities.

**Table 1:** Recommendations for future research on Voice Assistants (VAs) in heterogeneous groups of older adults.

	**Participatory design elements**	**Field studies**
Aims	•Conception and implementation of user training and manuals for using VAs together with the target group(s)	•Collect ecologically valid information on user experience •Gain insights into real-life use (and non-use) of VAs
Methods and design	•Co-designing, e.g., design workshops, prototype evaluation •Semi-structured interviews •Focus group discussions	•Diary studies, including cultural probes •Automatically collected data on usage (i.e., back-end data, audio- or video recordings) •Emotion analysis based on video and audio data
Expected outcomes and synergies	•Introduce VA use, benefits as well as limits and risks of VAs to different groups of older adults •Address common challenges of VAs for users with different levels of competences	•Get insights into user experiences beyond verbal and subjective feedback •Assess potentials of VAs for different groups •Improve user training and manuals

### Need for Participatory Design Elements in Research With Older Adults

We recommend a high level of user involvement in research about commercial VAs for older people. A participatory design strategy should identify the best ways to introduce VA use, benefits as well as limits and risks of VAs to different groups of older adults. In particular, this allows to address the identified challenges (see above) and may ensure that older people can use VAs according to their preferences. We argue that the need for instruction is higher among older adults compared to younger people because they did not grow up with digital technologies. As an example, we propose a participatory conception and implementation of user trainings and manuals^1^: To realize a participatory design in this domain, older adults should be involved in different co-designing activities such as co-design workshops to develop and discuss own ideas (e.g., Davidson and Jensen, 2013). Thereby, older users will be able to directly and actively influence the design of different kinds of material:

- *User trainings:* Educational programs and training courses can lower barriers in VA interaction (Czaja et al., 2019). The contents and format should be developed together with the target group and tailored to varying prior technology experiences, disability, and cognitive competencies. Relevant aspects may include the requirements for a successful interaction with VAs (e.g., specific voice commands), the possibilities of using VAs for different purposes (e.g., social domain, health, leisure time), and possible concerns (e.g., privacy issues, losing competencies due to using VAs). We recommend including older users with different skill levels in the training conception to make VAs widely accessible. Due to the different competencies (e.g., in reading and writing, attentional control, executive functions), the presentation and complexity must be adapted individually in each case.
- *User manuals:* Another aspect of VA learning and adoption are user manuals, an aspect that has so far been insufficiently addressed in research. Well-designed guidelines could help older adults to explore the possibilities of VAs according to their needs and in their own pace. Different versions of user manuals should be discussed with older adults to achieve the best design possible. These group-specific manuals are especially helpful for older adults with cognitive impairments or intellectual disabilities who may have specific needs, like instructions in easy-to-read language (Maaß, 2020) or a visualization of the instructions (Spriggs et al., 2017).

### Need for Field Studies in the Everyday Lives of Older Adults

Field studies can contribute to a high level of *ecological validity* of the research–implying the empirical validity of findings in everyday life settings. Depending on the research questions, researchers should thoroughly consider the appropriate observation period. Existing field studies about VA use report durations from only a few days (Lopatovska et al., 2019), several months (Scherr et al., 2020), up to 1 year (Trajkova and Martin-Hammond, 2020). Our recommendation would be an observation period of at least 4 weeks, which would allow analysis of more routinized interactions with VAs after an initial phase of learning and curiosity.

Despite existing field studies, we claim that the potentials are not yet fully exploited, and should be extended focusing on innovative approaches and heterogeneous user groups:

- *Diary studies*: In open-ended and closed questions, the users can report their experiences, likes, and dislikes about using a VA. A digital diary format allows to provide additional assistance if necessary. Participants can be reminded of the diary with prompts, and questions can be repeated and adapted to the individual. Participants can be actively encouraged to provide comprehensive feedback on enjoyable, useful, and negative experiences of VA interactions. In addition to this, cultural probes such as self-taken photos, cards with reflection tasks about VA use, and other activities (e.g., creation of relationship or neighborhood maps) can be applied to gain further insights into the everyday lives of the participants and to capture older adults' experiences with the devices in a comprehensive manner (Jarke and Gerhard, 2017).
- *Analysis of automatically collected data*: Beyond users' self-reports, we see high potential in collecting additional data associated with usage behaviors like back-end data combined with external recordings of audio (Porcheron et al., 2018) or video (Lahoual and Frejus, 2019) data of VA use. These data provide information about which VA functions are used by individuals with different levels of expertise and competences, about used voice commands, and changes of use patterns over time. In this context, researchers should in any case consider ethical concerns of automatically collected data such as a threat of permanent observation or the fear of providing too intimate information. Attention should be paid to the design of the informed consent and continuous support of study participants concerning these aspects should be provided.
- *Emotion analysis:* State-of-the-art software solutions allow to automatically analyze emotional experiences based on speech and facial expressions (Garcia-Garcia et al., 2017; Dupré et al., 2018). The emotional experience of VA use is another aspect that is important to our understanding of their benefits and challenges for older adults. An analysis of these data allows researchers to study user experiences of VAs in situations when verbal feedback is scarce due to possible cognitive impairments or intellectual disabilities. Still, due to the novelty of the approach, automatic emotion analysis is not always reliable. Regarding older adults with intellectual disabilities, their emotional expression may differ from older adults without disabilities due to more frequent motor impairments (e.g., spasticity), differences in physical appearance (especially regarding genetic syndromes), cognitive deficits (e.g., in perception or appraisal processes), stereotypical behaviors, or earlier aging processes (von Gontard, 2013). Facial expression may be altered so that automatic face recognition cannot detect known patterns. The validity of automatic emotion analysis in this group has not yet been proven (Adams and Oliver, 2011; Martínez-González and Veas, 2019) and should be the focus of future research.

In particular, the triangulation of the different data sources can provide an overarching picture of user experiences of VAs, e.g., by analyzing in which situations the VA is used, how the older person evaluates this interaction, and how it is experienced emotionally.

## Conclusions

In this opinion paper, we offer a set of recommendations that may guide future research on VAs for and with older adults. In a nutshell, we posit that future research designs should strongly rely on analyzing VA interactions in everyday ecologies and strictly apply participatory design elements where possible. Data protocols should include a balanced mixture of automatized data including emotional aspects as well as structured and open assessments. From our perspective, considering these recommendations can significantly help to create evidence-based findings able to inform interventions with VAs in heterogeneous groups of older adults. This also contributes to the goal of getting the best out of VA systems to improve quality of life and avoid possible risks.

## Author Contributions

All authors provided substantial contributions to this article from conception to final approval.

## Conflict of Interest

The authors declare that the research was conducted in the absence of any commercial or financial relationships that could be construed as a potential conflict of interest.

## Footnotes

^1^We put emphasis on commercial VAs that are available “off the shelf” but offer lower customization and co-designing options. However, in the case of *VA design and development*, participatory research and co-designing is equally important to better understand users' needs and their preferences. A co-design of VAs will guarantee that style, content, wording and tasks of the VAs will fit the mental models of older adults (Wolters et al., 2016; Fischer et al., 2020).
